# Nutrition, Gut Microbiota, and Alzheimer's Disease

**DOI:** 10.3389/fpsyt.2021.712673

**Published:** 2021-08-05

**Authors:** Mariana Romanenko, Victor Kholin, Alexander Koliada, Alexander Vaiserman

**Affiliations:** ^1^Laboratory of Dietetics, D.F. Chebotarev State Institute of Gerontology NAMS of Ukraine, Kyiv, Ukraine; ^2^Department of Age Physiology and Pathology of the Nervous System, D.F. Chebotarev State Institute of Gerontology NAMS of Ukraine, Kyiv, Ukraine; ^3^Molecular Genetic Laboratory Diagen, Kyiv, Ukraine; ^4^Laboratory of Epigenetics, D.F. Chebotarev State Institute of Gerontology NAMS of Ukraine, Kyiv, Ukraine

**Keywords:** nutrition, gut microbiota, cognitive function, dementia, Alzheimer's disease

## Abstract

Nutrition is known to play an important role in the pathogenesis of Alzheimer's disease. Evidence is obtained that the gut microbiota is a key player in these processes. Dietary changes (both adverse and beneficial) may influence the microbiome composition, thereby affecting the gut-brain axis and the subsequent risk for Alzheimer's disease progression. In this review, the research findings that support the role of intestinal microbiota in connection between nutritional factors and the risk for Alzheimer's disease onset and progression are summarized. The mechanisms potentially involved in these processes as well as the potential of probiotics and prebiotics in therapeutic modulation of contributed pathways are discussed.

## Introduction

Over 50 million people worldwide were living with dementia in 2019 and their number is expected to rise to 152 million in 2050 (1). Dementia, particularly Alzheimer's disease (AD), as the major cause of disability and dependence in elderly persons lead to a significant negative socioeconomic impact (2). In 2015, the global costs of dementia increased to 818 billion of US dollars and estimated costs in 2030 can reach about 2 trillion US dollars (3). Among the multiple risk factors identified for AD the presence of potentially modified cardiometabolic risk factors opens the opportunities to impact them through dietary modification (4, 5). Moreover, recent evidence indicates that imbalances in the gut microbiota (GM) can be also associated with the neurodegeneration (6, 7). So, the GM appears to be an attractive aim for prevention or treatment of AD (8–10). In this context, modulation of the GM composition offers diet a strong potential (6, 11).

## Nutrition and Alzheimer's Disease

In the last decades, the influence of dietary factors on cognitive function has become the subject of active research in pre-clinical and clinical studies. Various nutritional approaches have demonstrated a potential impact to prevent or slow down neurodegeneration or improve certain cognitive capacities. Accordingly, some benefits for cognitive performance may be found for vitamins E, D, B-group vitamins, various polyphenols, carotenoids, capsaicin, n-3 polyunsaturated fatty acids (PUFAs) and monosaturated fatty acids (MUFAs), some food and dietary patterns (12, 13).

### Food Group

The relationships between the consumption of various food groups and cognitive health has been investigated for many years. Currently, studies of the various foods impact on cognitive performance mostly report mixed data. Vegetable intake was correlated with better cognitive score in a prospective cohort study or with larger cortical thickness in a cross-sectional study (14, 15). Consumption of vegetables was also shown to improve orientation ability in cognitively healthy adults or adults with mild cognitive impairment (MCI) (16). Positive impact of vegetables on cognitive performance is thought to be attributed to high content of carotenoids, polyphenols, other antioxidants and fiber.

Animal protein food is of interest as it is a significant source for the formation of neurotransmitters or neurotoxic substances by the GM (17). However, fish consumption seems to affect cognitive function positively. In cognitively healthy elderly individuals higher fish intake was associated with larger cortical thickness or larger total gray matter volume (15, 18). Elderly adults with fish consumption ≥1 servings/week had a slower rate of cognitive decline (19). Nevertheless, in other study no evidence was found for fish to prevent age-related cognitive impairment (20). In meta-analysis by Bakre et al. a 20–30% decrease in the risk of dementia and AD in people who eat fish was reported (21). The potential benefits of fish intake are considered to be linked to the n*-*3 PUFAs content in marine fish (12). N-3 PUFAs were shown to diminish amyloid-beta (Aβ) peptide aggregation, increase Aβ clearance, modulate synaptic plasticity and Tau phosphorylation, and decrease neuroinflammation (22–26). Existing findings indicate that n-3 PUFAs may also influence the GM composition and intestinal barrier integrity (27, 28). Interestingly, cognitive changes induced by dietary n-3 PUFAs deficiency correlated with microbiota composition and inflammatory status in an animal study (29). However, in the systematic review by Rangel-Huerta et al. evidence that n-3 PUFAs supplementation can prevent or slow down AD in older adults appeared to be inconclusive (30).

Consumption of dairy and meat is thought to impact negatively on cognition, as a high intake of this protein sources is part of the unhealthy Western-style diet. Nonetheless, Ngabirano et al. found that elderly people who consumed meat ≤1 time/week were at an increased AD risk. Moreover, low meat consumers also ate less fish, fruit and vegetables, therefore, they could have low dietary intake in general and some nutritional deficiencies (31). No strong association between meat consumption and cognitive decline was observed in the meta-analysis by Zhang et al. (32).

Currently, several reviews failed to find a dose-response effect of milk and dairy consumption on cognitive performance (33, 34). In part, the mixed findings can be explained by methodological heterogeneity of studies included and the existence of opposing consumption patterns in countries with different dairy cultures such as Japan and the US, for example (35, 36). Concerns about dairy consumption are related to their D-galactose content since excess D-galactose has been shown to impair neuronal function (37). Interestingly, interventions with probiotics (38) or antioxidants (39, 40) may attenuate D-galactose-induced brain senescence in animal studies.

### Dietary Pattern

Accumulating evidence indicates that combinations of foods and nutrients might have a synergistic effect and thus more benefits on cognitive function than individual components. This may be due to the improved micronutrient intake and overall health and, of course, better microbiota composition in people adherent to healthy diet. Some dietary patterns such as Mediterranean diet (MeD), Dietary Approaches to Stop Hypertension (DASH) and Mediterranean-DASH Intervention for Neurodegenerative Delay (MIND) were associated with improved cognitive scores in population aged ≥40 y (12, 41, 42). MeD, DASH and MIND are plant-based diets with moderate to high consumption of fish, whole grains, vegetables and fruit, nuts and limited amount of red meat and sweets. Some differences between these diets lie in the amounts of other foods and nutrients (41–44). Higher adherence to MeD was associated with lower brain atrophy in non-demented elderly adults (15, 18). In the meta-analysis by van den Brink et al. higher MeD adherence decreased AD risk in case-control and longitudinal studies (42). The longitudinal studies showed a lower AD risk for high adherence to the DASH and MIND diets and for moderate adherence to the MIND diet (45, 46). Of note, the mentioned dietary patterns are high in fiber promoting the growth of healthy GM. Moreover, these diets contain nutrients associated with antioxidant and anti-inflammatory properties and suppression of Aβ deposition, including n-3 PUFAs, vitamin E, folate, carotenoids, and polyphenols (44, 47). Elevated MUFAs consumption as part of MeD and MIND diets is also considered to be beneficial for reducing the dementia risk (12, 48).

## Gut Microbiota Composition and Alzheimer's Disease

The previous data showed that the detrimental changes in the GM composition result in increase of intestinal permeability and systemic inflammation, which negatively affects the blood-brain barrier integrity (49–51). Further, bacterial lipopolysaccharides (LPS) and proinflammatory cytokines may activate microglia and accelerate neuroinflammation which contributes to a neuronal loss (7, 52–54). Activation of intestinal NLRP3 by gut flora was also shown to be involved into AD pathogenesis. In animal model upregulation of NLRP3 inflammasome after fecal microbiota transplantation (FMT) from AD patients lead to activation of systemic inflammation and neuroinflammation in the hippocampus (55). The most discussed potential mechanisms of GM impact on AD risk are associated with active metabolites and signaling molecules such as: trimethylamine N-oxide (TMAO) (56, 57), bile acids (58, 59), dysregulated P-glycoprotein (60), kynurenine (61), and nuclear factor-κB-sensitive microRNA-146a (62).

In addition, bacterial amyloid proteins prime the immune system, thus triggering an immune response to the brain amyloids and promoting the alpha-synuclein aggregation (63, 64). Therefore, an increasing number of studies are devoted to the relationship between AD and GM. Significant changes in the GM were demonstrated in many studies on AD mouse models. For example, 27 bacterial species from six phylogenetic groups differed in SAMP8 mice compared to the control (65). Significant differences in the GM composition were also revealed for AD patients, both at the phylum and species levels. Quantitative differences between AD patients and healthy participants were found in 13 bacterial genera. In particular, AD patients showed an increase in the number of bacteria belonging to *Proteobacteria* and *Bacteroidetes* phyla, together with a decrease in the representatives of *Firmicutes* and *Actinobacteria* phyla. Additionally, researchers demonstrated an association between the presence of some genera in the gut and AD markers, such as Aβ42/Aβ40 and Aβ/p-tau ratio (66). Noted remarkable differences in the bacterial diversity in the AD patients' intestines compared to healthy people were revealed in terms of taxonomic groups such as *Bacteroides, Actinobacteria, Ruminococcus, Lachnospiraceae*, and *Selenomonadales* (67). An increase of genera *Escherichia–Shigella*, a member of *Enterobacteriaceae*, and reduction of SCFA-producing genera was found by Hou et al. (68). In another study, AD patients showed higher prevalence of proinflammatory taxa and lower abundance of butyrate-producing species, such as members of the *Butyrivibrio* and *Eubacterium* genera, as well as *Clostridium sp*. strain SY8519, *Roseburia hominis*, and *Faecalibacterium prausnitzii* (60). Notably, infection with certain pathogens including representatives of the oral or gut microbiome such as *Helicobacter pylori, Porphyromonas gingivalis, Candida albicans, Candida glabrata*, and others were found to be associated with AD risk (69).

Due to obvious differences in the microbiome composition, the first attempts to use microbial signatures as markers of AD are being made (70). For example, a model that uses 20 typical predominant genera can effectively distinguish patients with AD and MCI from healthy individuals. Moreover, five functional orthologs which differed in AD patients were identified by using metagenomic data. The samples obtained from AD patients showed a deficit of orthologs engaged into the biosynthesis of the amino acids involved in the metabolism of neurotransmitters (71). These results are in line with other studies which revealed the dysregulation of tryptophan metabolic pathways in AD patients (72, 73).

## Microbiota-Mediated Link Between Nutrition and Alzheimer's Disease

**Figure 1:**
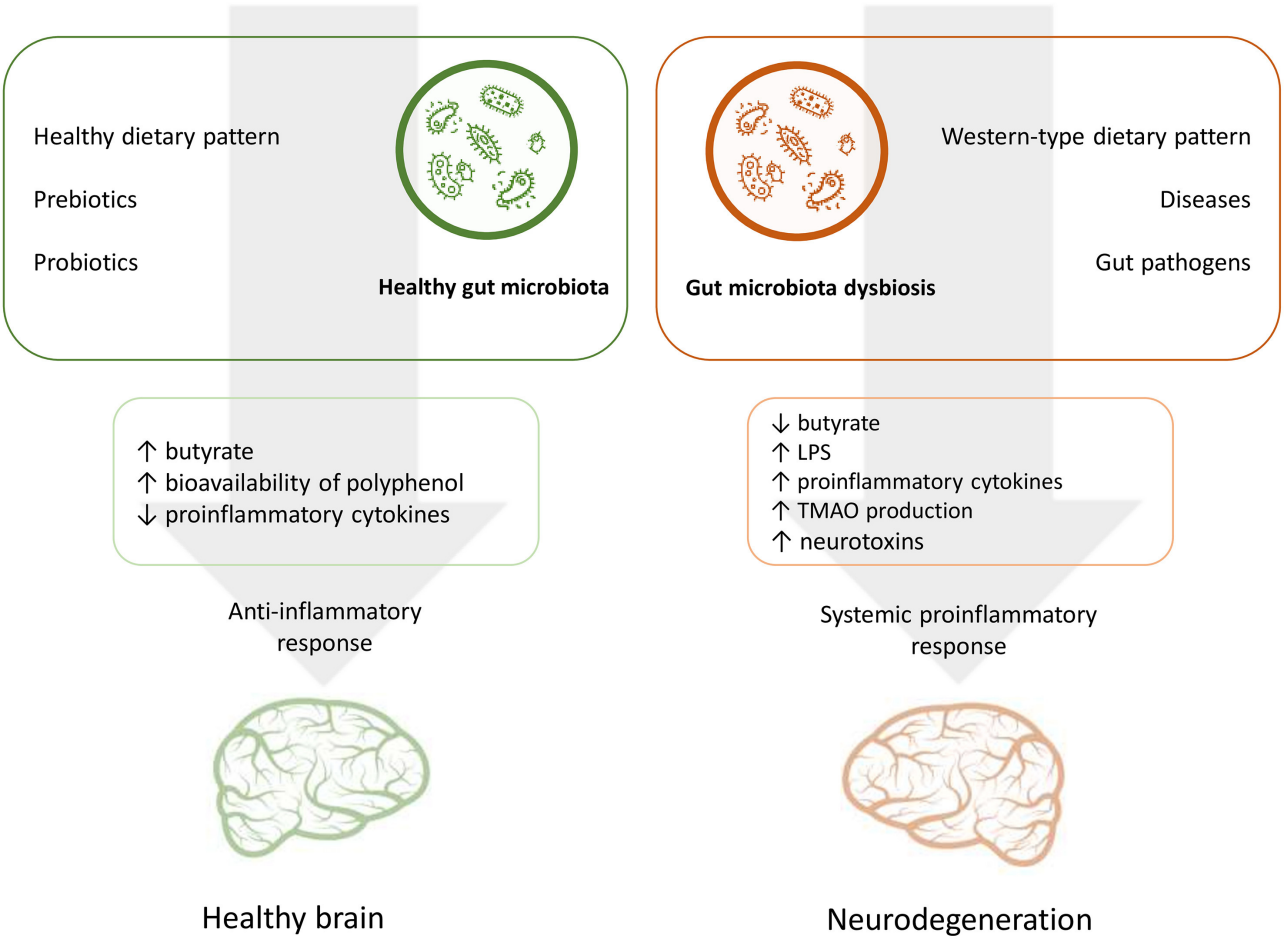
The role of nutrition and gut microbiota in the pathogenesis of Alzheimer's disease.

Nutrition is one of the main factors that influence the GM composition throughout the life course (74, 75). In turn, microbiota mediate interplay between habitual diet and various processes of a host organism, including cognitive performance (6, 10). In this context the GM may interact with dietary factors by contributing to energy homeostasis and metabolic risk factors and modulating systemic inflammatory response through dietary metabolites and also affecting the availability of nutrients which are important for brain functioning (49, 50, 52, 76). Figure 1 summarizes the potential effects of nutrition and the GM on the AD risk.

### Short-Chain Fatty Acids

A positive impact of GM on neuronal homeostasis is associated with short-chain fatty acids (SCFAs) derived from non-digested polysaccharides (77). SCFAs were shown to affect brain functions directly by improving blood-brain barrier integrity and affecting glial cells or indirectly by modulating the immune response, and activating vagal and humoral pathways of the gut-brain axis (78–81). SCFAs such as acetate, propionate, and butyrate regulate many cellular functions via binding to G-protein-coupled receptors (82). Moreover, acetate and butyrate are also well-known inhibitors of histone deacetylases, which activity is associated with cellular aging (83, 84). Anti-inflammatory and neuroprotective capacities of SCFAs were found mainly in animal studies or *in vitro* (85–87). In contrast, in one study increased Aβ plaque deposition was demonstrated in germ free AD mice fed with SCFAs (88). In human studies acetate and valerate as well as bacterial LPS were connected with an enhanced amyloid deposition in the brain. Conversely, a high serum level of butyrate was found to be associated with fewer amyloid plaques (89).

### Polyphenols

Current findings indicate that GM closely interacts with dietary polyphenols using them as a food source for its own growth and providing new microbiota-derived metabolites. Thus, dietary polyphenols were shown to promote growth of *Lactobacillus spp*. and *Bifidobacterium spp*. and inhibited potentially pathogenic species (90, 91). On the other hand, GM potentially enhances the bioavailability of phenolic metabolites to the host (92, 93). Benefits of polyphenols and their metabolites in prevention of cognitive decline associated with its anti-inflammatory properties were reported previously (94–97). For example, gallic acid, which is a bacterial-derived metabolite of anthocyanins, was shown to decrease Aβ deposition, reduce neuroinflammation and oxidative stress in brain of AD mice (98). Noteworthy that polyphenols and their metabolites may enhance the intestinal barrier integrity and thus decrease the local and systemic inflammation (99–101). Accordingly, the GM activity contributes to cognitive promotion effect of polyphenol-rich dietary patterns like MeD, DASH, and MIND. Particularly, a positive association between certain phenolic compounds and abundance of a butyrate-producing *Faecalibacterium prausnitzii* was found in healthy adults adherent to MeD (102).

### Trimethylamine-N-oxide

TMAO, which is a bacterial-derived metabolite of dietary choline, betaine and l-carnitine, was shown to be related to cognitive decline and AD (56, 57). An increased TMAO level in cerebrospinal fluid was found in individuals with MCI as well as with AD. Moreover, elevated TMAO in the cerebrospinal fluid was associated with markers of neurodegeneration (57). It is suggested that the TMAO blood level may depend on various factors, including the diet, GM composition, liver enzymes activity and urinary excretion (103, 104). However, the interaction between TMAO, its precursors, and neurodegeneration remains not fully understood. All of TMAO dietary substrates were previously found to be beneficial for the cognitive function (105–110). Systematic reviews and meta-analyses elucidating the impact on cognition of food rich in TMAO precursors such as meat, eggs, dairy products, and marine fish demonstrated mixed results, although fish intake seemed to improve cognitive performance (12, 21, 32–34). Interestingly, marine fish already contains an increased amount of TMAO (103, 111). Studies with food or supplements rich in TMAO precursor failed to increase fasting plasma TMAO in young healthy adults (112, 113). Despite this, a concomitant increase in plasma choline, betaine and gamma-butyrobetaine was observed in study by Berge et al. (112). Switching to the MeD did not affect fasting TMAO, choline, betaine, and carnitine in healthy adults with an increased colon cancer risk (114). However, in another study daily red meat consumption (meat protein was 12% of daily energy) elevated TMAO concentration in plasma and urine. Moreover, red meat intake decreased TMAO renal excretion (115). Thus, it remains unclear whether consumption of TMAO precursors should be restricted for better cognitive performance.

Current findings showed that the impact of microbiota on TMAO metabolism may be related to an abundance of strains producing trimethylamine (TMA). Among the TMA-producing species belonging to *Firmicutes* and *Proteobacteria* phyla the representatives of *Clostridium, Escherichia*, and *Proteus* genera were identified (116). It is notable that cognitive impaired patients with Aβ deposition had higher abundance of the genus *Escherichia/Shigella* as compared to Aβ negative patients and healthy controls (117). In other studies, healthy individuals with a higher *Firmicutes* to *Bacteroidetes* ratio demonstrated an increased production of TMAO from choline and carnitine rich food (118, 119). Negative correlation was found between the *Akkermansia mucinophilia* presence and fasting plasma TMAO in healthy adults at risk for colon cancer (114). Additionally, the GM was shown to regulate TMAO production via the impact on converting enzyme activity in the liver (117). However, in other neurodegenerative diseases lower plasma TMAO was reported to have worse prognostic implications (120, 121). Altogether, the existing data indicate that the interaction between TMAO, its precursors and cognitive impairment is more complex than a direct link and GM may play in it one of the key roles.

### Prebiotics and Probiotics

The modulation of systemic inflammatory response with prebiotics and probiotics can be part of a comprehensive approach to slow down the cognitive decline through an impact on gut-brain axis (122, 123). A positive effect on cognitive performance for diets rich in fiber and polyphenols can partially be attributed to the prebiotic properties of the mentioned nutrients. Studies on AD mouse models revealed neuroprotective effects for some non-digested fermentable carbohydrates, such as mannan oligosaccharide (124), *Morinda officinalis* oligosaccharides (125), xylooligosaccharides (126), yeast β-glucans (127), lactulose and trehalose (128). Currently, human studies of prebiotics impact on cognitive function in AD patients are scarce.

Animal studies on probiotics showed that feeding with *Bifidobacterium breve* A1 restored the impaired cognitive behavior and suppressed neuroinflammation in the hippocampus of AD mice (129). Human interventions with *Bifidobacteria spp*. revealed an improvement in cognitive scores under up to 6 months supplementation in elderly adults with MCI (130, 131). The interventions with probiotics in AD individuals as of today are quite limited but still promising. Shot-term supplementation with multispecies probiotic did not change cognitive scores in AD patients but affected the microbiome leading to an increase in *Faecalibacterium prausnitzii* and decreased intestinal permeability. A concomitant increase in serum neopterin and tryptophan breakdown marker could indicate the stimulation of the immune system (132). In small research of supplementation with probiotic-fermented milk AD individuals demonstrated a decrease in inflammatory and oxidative responses, reduction of DNA damage and apoptosis in peripheral blood leucocytes (133). In another study, probiotic stains plus selenium supplementation resulted in a better cognitive score in AD patients. Moreover, probiotics enhanced the selenium effect on the reduction of high sensitive C-reactive protein and an increase in total antioxidant capacity and the glutathione level (134).

## Fecal Microbiota Transplantation

FMT is a transfer of fecal material from a healthy donor into the patient's gastrointestinal tract. This procedure is a powerful means of regulating the GM and is being actively studied for many diseases at the moment. Despite this approach being promising, we have very limited data on the use of this method for AD (135). FMT from WT mice into ADLP^APT^ mice improved intestinal barrier integrity and decreased the formation of amyloid plaques and neurofibrillary tangles in animal brains (136). In another AD mouse model FMT from healthy individual following FMT from AD patients decreased the expression of pro-inflammatory cytokines in blood and hippocampus and improved cognitive ability of animals (55). One case study reported long-term improvement in the mental acuity of 82-year-old patient after FMT (137).

## Conclusion

Existing evidence suggests that dietary lifestyle changes may affect cognitive function. Certain nutrients appear to be beneficial in maintaining neuronal homeostasis and slowing cognitive decline. Along with it, the most convincing evidence was reported for whole-diet approaches such as MeD, DASH, and MIND. One of the key roles in the diet impact on cognitive performance and AD risk belongs to the composition and functional activity of the GM. It has now been shown that microbiota affects brain functions through various metabolites with potentially positive or, conversely, toxic properties. Moreover, by converting food precursors, the intestinal flora regulates the availability of nutrients important for cognition. It seems that the GM involvement in the SCFA and polyphenols metabolism may contribute to the cognitive promotion effect of healthy dietary patterns. In addition, the use of probiotics can be part of a comprehensive approach to delay neurodegeneration. However, for a long-term GM modification, a whole-diet may have advantages over nutrients alone or in combination. Overall, the GM is an important factor to be considered in future research of dietary effects on cognitive function. Thus, the underlying mechanisms of interaction between nutrition, microbiota, and the host require additional studies for developing effective dietary strategies within integrated AD prevention and control.

## Author Contributions

AV conceptualized the structure and wrote the first draft. MR, AK, and VK co-wrote the first draft. MR edited the final version of the manuscript. All authors approved the final version of the manuscript.

## Conflict of Interest

The authors declare that the research was conducted in the absence of any commercial or financial relationships that could be construed as a potential conflict of interest.

## Publisher's Note

All claims expressed in this article are solely those of the authors and do not necessarily represent those of their affiliated organizations, or those of the publisher, the editors and the reviewers. Any product that may be evaluated in this article, or claim that may be made by its manufacturer, is not guaranteed or endorsed by the publisher.

## References

[1] Alzheimer's Disease International. World Alzheimer Report 2019: Attitudes to dementia. London: Alzheimer's Disease International (2019). p. 166. Available online at: https://www.alzint.org/u/WorldAlzheimerReport2019.pdf (accessed May 2, 2021).

[2] El-Hayek YH Wiley RE Khoury CP Daya RP Ballard C Evans AR. Tip of the Iceberg: assessing the global socioeconomic costs of Alzheimer's disease and related dementias and strategic implications for stakeholders. J Alzheimers Dis. (2019) 70:323–41. 10.3233/JAD-190426 31256142 PMC6700654

[3] Wimo A Guerchet M Ali GC Wu YT Prina AM Winblad B. The worldwide costs of dementia 2015 and comparisons with 2010. Alzheimers Dement. (2017) 13:1–7. 10.1016/j.jalz.2016.07.150 27583652 PMC5232417

[4] Vauzour D Camprubi-Robles M Miquel-Kergoat S Andres-Lacueva C Bánáti D Barberger-Gateau P. Nutrition for the ageing brain: towards evidence for an optimal diet. Ageing Res Rev. (2017) 35:222–40. 10.1016/j.arr.2016.09.010 27713095

[5] Scarmeas N Anastasiou CA Yannakoulia M. Nutrition and prevention of cognitive impairment. Lancet Neurol. (2018) 7:1006–15. 10.1016/S1474-4422(18)30338-7 30244829

[6] Pistollato F Sumalla Cano S Elio I Masias Vergara M Giampieri F Battino M. Role of gut microbiota and nutrients in amyloid formation and pathogenesis of Alzheimer disease. Nutr Rev. (2016) 74:624–34. 10.1093/nutrit/nuw023 27634977

[7] Pellegrini C Antonioli L Colucci R Blandizzi C Fornai M. Interplay among gut microbiota, intestinal mucosal barrier and enteric neuro-immune system: a common path to neurodegenerative diseases? Acta Neuropathol. (2018) 136:345–61. 10.1007/s00401-018-1856-5 29797112

[8] Sampson TR Mazmanian SK. Control of brain development, function, and behavior by the microbiome. Cell Host Microbe. (2015) 17:565–76. 10.1016/j.chom.2015.04.011 25974299 PMC4442490

[9] Cerovic M Forloni G Balducci C. Neuroinflammation and the gut microbiota: possible alternative therapeutic targets to counteract Alzheimer's disease? Front Aging Neurosci. (2019) 11:284. 10.3389/fnagi.2019.00284 31680937 PMC6813195

[10] Shabbir U Arshad MS Sameen A Oh DH. Crosstalk between gut and brain in Alzheimer's disease: the role of gut microbiota modulation strategies. Nutrients. (2021) 13:690. 10.3390/nu13020690 33669988 PMC7924846

[11] García-Montero C Fraile-Martínez O Gómez-Lahoz AM Pekarek L Castellanos AJ Noguerales-Fraguas F. Nutritional components in Western diet versus Mediterranean diet at the gut microbiota-immune system interplay. Implications for health and disease. Nutrients. (2021) 13:699. 10.3390/nu13020699 33671569 PMC7927055

[12] Solfrizzi V Custodero C Lozupone M Imbimbo BP Valiani V Agosti P. Relationships of dietary patterns, foods, and micro- and macronutrients with Alzheimer's disease and late-life cognitive disorders: a systematic review. J Alzheimers Dis. (2017) 59:815–49. 10.3233/JAD-170248 28697569

[13] Moore K Hughes CF Ward M Hoey L McNulty H. Diet, nutrition and the ageing brain: current evidence and new directions. Proc Nutr Soc. (2018) 77:152–63. 10.1017/S0029665117004177 29316987

[14] Trichopoulou A Kyrozis A Rossi M Katsoulis M Trichopoulos D La Vecchia C. Mediterranean diet and cognitive decline over time in an elderly Mediterranean population. Eur J Nutr. (2015) 54:1311–21. 10.1007/s00394-014-0811-z 25482573

[15] Staubo SC Aakre JA Vemuri P Syrjanen JA Mielke MM Geda YE. Mediterranean diet, micronutrients and macronutrients, and MRI measures of cortical thickness. Alzheimers Dement. (2017) 13:168–77. 10.1016/j.jalz.2016.06.2359 27461490 PMC5259552

[16] Dong L Xiao R Cai C Xu Z Wang S Pan L. Diet, lifestyle and cognitive function in old Chinese adults. Arch Gerontol Geriatr. (2016) 63:36–42. 10.1016/j.archger.2015.12.003 26791169

[17] Oliphant K Allen-Vercoe E. Macronutrient metabolism by the human gut microbiome: major fermentation by-products and their impact on host health. Microbiome. (2019) 7:91. 10.1186/s40168-019-0704-8 31196177 PMC6567490

[18] Gu Y Brickman AM Stern Y Habeck CG Razlighi QR Luchsinger JA. Mediterranean diet and brain structure in a multiethnic elderly cohort. Neurology. (2015) 85:1744–51. 10.1212/WNL.0000000000002121 26491085 PMC4653103

[19] Qin B Plassman BL Edwards LJ Popkin BM Adair LS Mendez MA. Fish intake is associated with slower cognitive decline in Chinese older adults. J Nutr. (2014) 144:1579–85. 10.3945/jn.114.193854 25080536 PMC4162477

[20] Fischer K Melo van Lent D Wolfsgruber S Weinhold L Kleineidam L Bickel H. Prospective associations between single foods, Alzheimer's dementia and memory decline in the elderly. Nutrients. (2018) 10:852. 10.3390/nu10070852 29966314 PMC6073331

[21] Bakre AT Chen R Khutan R Wei L Smith T Qin G. Association between fish consumption and risk of dementia: a new study from China and a systematic literature review and meta-analysis. Public Health Nutr. (2018) 21:1921–32. 10.1017/S136898001800037X 29551101 PMC10260768

[22] Fiala M Kooij G Wagner K Hammock B Pellegrini M. Modulation of innate immunity of patients with Alzheimer's disease by omega-3 fatty acids. FASEB J. (2017) 31:3229–39. 10.1096/fj.201700065R 28420693 PMC5503712

[23] Joffre C Rey C Layé S. N-3 polyunsaturated fatty acids and the resolution of neuroinflammation. Front Pharmacol. (2019) 10:1022. 10.3389/fphar.2019.01022 31607902 PMC6755339

[24] Morgese MG Schiavone S Maffione AB Tucci P Trabace L. Depressive-like phenotype evoked by lifelong nutritional omega-3 deficiency in female rats: crosstalk among kynurenine, Toll-like receptors and amyloid beta oligomers. Brain Behav Immun. (2020) 87:444–54. 10.1016/j.bbi.2020.01.015 31987923

[25] Fonteh AN Cipolla M Chiang AJ Edminster SP Arakaki X Harrington MG. Polyunsaturated fatty acid composition of cerebrospinal fluid fractions shows their contribution to cognitive resilience of a pre-symptomatic Alzheimer's disease cohort. Front Physiol. (2020) 11:83. 10.3389/fphys.2020.00083 32116789 PMC7034243

[26] Melo van Lent D Egert S Wolfsgruber S Kleineidam L Weinhold L Wagner-Thelen H. Eicosapentaenoic acid is associated with decreased incidence of Alzheimer's dementia in the oldest old. Nutrients. (2021) 13:461. 10.3390/nu13020461 33573174 PMC7912244

[27] Kaliannan K Wang B Li XY Kim KJ Kang JX. A host-microbiome interaction mediates the opposing effects of omega-6 and omega-3 fatty acids on metabolic endotoxemia. Sci Rep. (2015) 5:11276. 10.1038/srep11276 26062993 PMC4650612

[28] Cao W Wang C Chin Y Chen X Gao Y Yuan S. DHA-phospholipids (DHA-PL) and EPA-phospholipids (EPA-PL) prevent intestinal dysfunction induced by chronic stress. Food Funct. (2019) 10:277–88. 10.1039/C8FO01404C 30565622

[29] Robertson RC Seira Oriach C Murphy K Moloney GM Cryan JF Dinan TG. Omega-3 polyunsaturated fatty acids critically regulate behaviour and gut microbiota development in adolescence and adulthood. Brain Behav Immun. (2017) 59:21–37. 10.1016/j.bbi.2016.07.145 27423492

[30] Rangel-Huerta OD Gil A. Effect of omega-3 fatty acids on cognition: an updated systematic review of randomized clinical trials. Nutr Rev. (2018) 76:1–20. 10.1093/nutrit/nux064 29240924

[31] Ngabirano L Samieri C Feart C Gabelle A Artero S Duflos C. Intake of meat, fish, fruits, and vegetables and long-term risk of dementia and Alzheimer's disease. J Alzheimers Dis. (2019) 68:711–22. 10.3233/JAD-180919 30883348

[32] Zhang H Hardie L Bawajeeh AO Cade J. Meat consumption, cognitive function and disorders: a systematic review with narrative synthesis and meta-analysis. Nutrients. (2020) 12:1528. 10.3390/nu12051528 32456281 PMC7285210

[33] Lee J Fu Z Chung M Jang DJ Lee HJ. Role of milk and dairy intake in cognitive function in older adults: a systematic review and meta-analysis. Nutr J. (2018) 17:82. 10.1186/s12937-018-0387-1 30149812 PMC6112122

[34] Bermejo-Pareja F Ciudad-Cabañas MJ Llamas-Velasco S Tapias-Merino E Hernández Gallego J Hernández-Cabria M. Is milk and dairy intake a preventive factor for elderly cognition (dementia and Alzheimer's)? A quality review of cohort surveys. Nutr Rev. (2020) 14:nuaa045. 10.1093/nutrit/nuaa045 33316068

[35] Petruski-Ivleva N Kucharska-Newton A Palta P Couper D Meyer K Graff M. Milk intake at midlife and cognitive decline over 20 years. The Atherosclerosis Risk in Communities (ARIC) Study. Nutrients. (2017) 9:1134. 10.3390/nu9101134 29039795 PMC5691750

[36] Ozawa M Ohara T Ninomiya T Hata J Yoshida D Mukai N. Milk and dairy consumption and risk of dementia in an elderly Japanese population: the Hisayama Study. J Am Geriatr Soc. (2014) 62:1224–30. 10.1111/jgs.12887 24916840

[37] Shwe T Pratchayasakul W Chattipakorn N Chattipakorn SC. Role of D-galactose-induced brain aging and its potential used for therapeutic interventions. Exp Gerontol. (2018) 101:13–36. 10.1016/j.exger.2017.10.029 29129736

[38] Nimgampalle M Kuna Y. Anti-Alzheimer properties of probiotic, Lactobacillus plantarum MTCC 1325 in Alzheimer's disease induced albino rats. J Clin Diagn Res. (2017) 11:KC01–5. 10.7860/JCDR/2017/26106.10428 28969160 PMC5620801

[39] Banji OJ Banji D Ch K. Curcumin and hesperidin improve cognition by suppressing mitochondrial dysfunction and apoptosis induced by D-galactose in rat brain. Food Chem Toxicol. (2014) 74:51–9. 10.1016/j.fct.2014.08.020 25217884

[40] Wang C Cai Z Wang W Wei M Si X Shang Y. Piperine regulates glycogen synthase kinase-3β-related signaling and attenuates cognitive decline in D-galactose-induced aging mouse model. J Nutr Biochem. (2020) 75:108261. 10.1016/j.jnutbio.2019.108261 31710934

[41] Tsivgoulis G Judd S Letter AJ Alexandrov AV Howard G Nahab F. Adherence to a Mediterranean diet and risk of incident cognitive impairment. Neurology. (2013) 80:1684–92. 10.1212/WNL.0b013e3182904f69 23628929 PMC3716473

[42] van den Brink AC Brouwer-Brolsma EM Berendsen AAM van de Rest O. The mediterranean, dietary approaches to stop hypertension (DASH), and mediterranean-DASH intervention for neurodegenerative delay (mind) diets are associated with less cognitive decline and a lower risk of Alzheimer's disease-a review. Adv Nutr. (2019) 10:1040–65. 10.1093/advances/nmz054 31209456 PMC6855954

[43] Solfrizzi V Frisardi V Capurso C D'Introno A Colacicco AM Vendemiale G. Dietary fatty acids in dementia and predementia syndromes: epidemiological evidence and possible underlying mechanisms. Ageing Res Rev. (2010) 9:184–99. 10.1016/j.arr.2009.07.005 19643207

[44] Morris MC Tangney CC Wang Y Sacks FM Barnes LL Bennett DA. MIND diet slows cognitive decline with aging. Alzheimers Dement. (2015) 11:1015–22. 10.1016/j.jalz.2015.04.011 26086182 PMC4581900

[45] Morris MC Tangney CC Wang Y Sacks FM Bennett DA Aggarwal NT. MIND diet associated with reduced incidence of Alzheimer's disease. Alzheimers Dement. (2015) 11:1007–14. 10.1016/j.jalz.2014.11.009 25681666 PMC4532650

[46] Hosking DE Eramudugolla R Cherbuin N Anstey KJ. MIND not Mediterranean diet related to 12-year incidence of cognitive impairment in an Australian longitudinal cohort study. Alzheimers Dement. (2019) 15:581–89. 10.1016/j.jalz.2018.12.011 30826160

[47] Godos J Rapisarda G Marventano S Galvano F Mistretta A Grosso G. Association between polyphenol intake and adherence to the Mediterranean diet in Sicily, southern Italy. NFS Journal. (2017) 8:1–7. 10.1016/j.nfs.2017.06.001

[48] Zhang T Han X Zhang X Chen Z Mi Y Gou X. Dietary fatty acid factors in Alzheimer's disease: a review. J Alzheimers Dis. (2020) 78:887–904. 10.3233/JAD-200558 33074226

[49] Jiang C Li G Huang P Liu Z Zhao B. The gut microbiota and Alzheimer's disease. J Alzheimers Dis. (2017) 58:1–15. 10.3233/JAD-161141 28372330

[50] Askarova S Umbayev B Masoud AR Kaiyrlykyzy A Safarova Y Tsoy A. The links between the gut microbiome, aging, modern lifestyle and Alzheimer's disease. Front Cell Infect Microbiol. (2020) 10:104. 10.3389/fcimb.2020.00104 32257964 PMC7093326

[51] Le Page A Dupuis G Frost EH Larbi A Pawelec G Witkowski JM. Role of the peripheral innate immune system in the development of Alzheimer's disease. Exp Gerontol. (2018) 107:59–66. 10.1016/j.exger.2017.12.019 29275160

[52] Spielman LJ Gibson DL Klegeris A. Unhealthy gut, unhealthy brain: the role of the intestinal microbiota in neurodegenerative diseases. Neurochem Int. (2018) 120:149–63. 10.1016/j.neuint.2018.08.005 30114473

[53] Lin L Zheng LJ Zhang LJ. Neuroinflammation, gut microbiome, and Alzheimer's disease. Mol Neurobiol. (2018) 55:8243–50. 10.1007/s12035-018-0983-2 29524051

[54] Kowalski K Mulak A. Brain-gut-microbiota axis in Alzheimer's disease. J Neurogastroenterol Motil. (2019) 25:48–60. 10.5056/jnm18087 30646475 PMC6326209

[55] Shen H Guan Q Zhang X Yuan C Tan Z Zhai L. New mechanism of neuroinflammation in Alzheimer's disease: the activation of NLRP3 inflammasome mediated by gut microbiota. Prog Neuropsychopharmacol Biol Psychiatry. (2020) 100:109884. 10.1016/j.pnpbp.2020.109884 32032696

[56] Li D Ke Y Zhan R Liu C Zhao M Zeng A. Trimethylamine-N-oxide promotes brain aging and cognitive impairment in mice. Aging Cell. (2018) 17:e12768. 10.1111/acel.12768 29749694 PMC6052480

[57] Vogt NM Romano KA Darst BF Engelman CD Johnson SC Carlsson CM. The gut microbiota-derived metabolite trimethylamine N-oxide is elevated in Alzheimer's disease. Alzheimers Res Ther. (2018) 10:124. 10.1186/s13195-018-0451-2 30579367 PMC6303862

[58] Nho K Kueider-Paisley A MahmoudianDehkordi S Arnold M Risacher SL Louie G. Alzheimer's Disease Neuroimaging Initiative and the Alzheimer Disease Metabolomics Consortium. Altered bile acid profile in mild cognitive impairment and Alzheimer's disease: relationship to neuroimaging and CSF biomarkers. Alzheimers Dement. (2019) 15:232–44. 10.1101/284141 30337152 PMC6454538

[59] MahmoudianDehkordi S Arnold M Nho K Ahmad S Jia W Xie G. Alzheimer's Disease Neuroimaging Initiative and the Alzheimer Disease Metabolomics Consortium. Altered bile acid profile associates with cognitive impairment in Alzheimer's disease-an emerging role for gut microbiome. Alzheimers Dement. (2019) 15:76–92. 10.1016/j.jalz.2018.07.217 30337151 PMC6487485

[60] Haran JP Bhattarai SK Foley SE Dutta P Ward DV Bucci V. Alzheimer's disease microbiome is associated with dysregulation of the anti-inflammatory P-glycoprotein pathway. mBio. (2019) 10:e00632–19. 10.1128/mBio.00632-19 31064831 PMC6509190

[61] Garcez ML Jacobs KR Guillemin GJ. Microbiota alterations in Alzheimer's disease: involvement of the kynurenine pathway and inflammation. Neurotox Res. (2019) 36:424–36. 10.1007/s12640-019-00057-3 31089885

[62] Zhao Y Lukiw WJ. Microbiome-mediated upregulation of microRNA-146a in sporadic Alzheimer's disease. Front Neurol. (2018) 9:145. 10.3389/fneur.2018.00145 29615954 PMC5867462

[63] Friedland RP Chapman MR. The role of microbial amyloid in neurodegeneration. PLoS Pathog. (2017) 13:e1006654. 10.1371/journal.ppat.1006654 29267402 PMC5739464

[64] Chen SG Stribinskis V Rane MJ Demuth DR Gozal E Roberts AM. Exposure to the functional bacterial amyloid protein curli enhances alpha-synuclein aggregation in aged fischer 344 rats and Caenorhabditis elegans. Sci Rep. (2016) 6:34477. 10.1038/srep34477 27708338 PMC5052651

[65] Zhan G Yang N Li S Huang N Fang X Zhang J. Abnormal gut microbiota composition contributes to cognitive dysfunction in SAMP8 mice. Aging (Albany NY). (2018) 10:1257–67. 10.18632/aging.101464 29886457 PMC6046237

[66] Vogt NM Kerby RL Dill-McFarland KA Harding SJ Merluzzi AP Johnson SC. Gut microbiome alterations in Alzheimer's disease. Sci Rep. (2017) 7:13537. 10.1038/s41598-017-13601-y 29051531 PMC5648830

[67] Zhuang ZQ Shen LL Li WW Fu X Zeng F Gui L. Gut microbiota is altered in patients with Alzheimer's disease. J Alzheimers Dis. (2018) 63:1337–46. 10.3233/JAD-180176 29758946

[68] Hou M Xu G Ran M Luo W Wang H. APOE-ε4 carrier status and gut microbiota dysbiosis in patients with Alzheimer disease. Front Neurosci. (2021) 15:619051. 10.3389/fnins.2021.619051 33732104 PMC7959830

[69] Panza F Lozupone M Solfrizzi V Watling M Imbimbo BP. Time to test antibacterial therapy in Alzheimer's disease. Brain. (2019) 142:2905–29. 10.1093/brain/awz244 31532495

[70] Ticinesi A Nouvenne A Tana C Prati B Meschi T. Gut microbiota and microbiota-related metabolites as possible biomarkers of cognitive aging. Adv Exp Med Biol. (2019) 1178:129–54. 10.1007/978-3-030-25650-0_8 31493226

[71] Liu P Wu L Peng G Han Y Tang R Ge J. Altered microbiomes distinguish Alzheimer's disease from amnestic mild cognitive impairment and health in a Chinese cohort. Brain Behav Immun. (2019) 80:633–43. 10.1016/j.bbi.2019.05.008 31063846

[72] Wu L Han Y Zheng Z Peng G Liu P Yue S. Altered gut microbial metabolites in amnestic mild cognitive impairment and Alzheimer's disease: signals in host-microbe interplay. Nutrients. (2021) 13:228. 10.3390/nu13010228 33466861 PMC7829997

[73] Whiley L Chappell KE D'Hondt E Lewis MR Jiménez B Snowden SG. Metabolic phenotyping reveals a reduction in the bioavailability of serotonin and kynurenine pathway metabolites in both the urine and serum of individuals living with Alzheimer's disease. Alzheimers Res Ther. (2021) 13:20. 10.1186/s13195-020-00741-z 33422142 PMC7797094

[74] Flint HJ. The impact of nutrition on the human microbiome. Nutr Rev. (2012) 70:S10–3. 10.1111/j.1753-4887.2012.00499.x 22861801

[75] Quercia S Candela M Giuliani C Turroni S Luiselli D Rampelli S. From lifetime to evolution: timescales of human gut microbiota adaptation. Front Microbiol. (2014) 5:587. 10.3389/fmicb.2014.00587 25408692 PMC4219431

[76] Koh A De Vadder F Kovatcheva-Datchary P Bäckhed F. From dietary fiber to host physiology: short-chain fatty acids as key bacterial metabolites. Cell. (2016) 165:1332–45. 10.1016/j.cell.2016.05.041 27259147

[77] Blaak EE Canfora EE Theis S Frost G Groen AK Mithieux G. Short chain fatty acids in human gut and metabolic health. Benef Microbes. (2020) 11:411–55. 10.3920/BM2020.0057 32865024

[78] Braniste V Al-Asmakh M Kowal C Anuar F Abbaspour A Tóth M. The gut microbiota influences blood-brain barrier permeability in mice. Sci Transl Med. (2014) 6:263ra158. 10.1126/scitranslmed.3009759 25411471 PMC4396848

[79] Corrêa-Oliveira R Fachi JL Vieira A Sato FT Vinolo MA. Regulation of immune cell function by short-chain fatty acids. Clin Transl Immunology. (2016) 5:e73. 10.1038/cti.2016.17 27195116 PMC4855267

[80] Dalile B Van Oudenhove L Vervliet B Verbeke K. The role of short-chain fatty acids in microbiota-gut-brain communication. Nat Rev Gastroenterol Hepatol. (2019) 16:461–78. 10.1038/s41575-019-0157-3 31123355

[81] Silva YP Bernardi A Frozza RL. The role of short-chain fatty acids from gut microbiota in gut-brain communication. Front Endocrinol (Lausanne). (2020) 11:25. 10.3389/fendo.2020.00025 32082260 PMC7005631

[82] Samuel BS Shaito A Motoike T Rey FE Backhed F Manchester JK. Effects of the gut microbiota on host adiposity are modulated by the short-chain fatty-acid binding G protein-coupled receptor, Gpr41. Proc Natl Acad Sci USA. (2008) 105:16767–72. 10.1073/pnas.0808567105 18931303 PMC2569967

[83] Soliman ML Rosenberger TA. Acetate supplementation increases brain histone acetylation and inhibits histone deacetylase activity and expression. Mol Cell Biochem. (2011) 352:173–80. 10.1007/s11010-011-0751-3 21359531

[84] Vaiserman AM Koliada AK Koshel' NM Simonenko AV Pasiukova EG. [Effect of the histone deacetylase inhibitor sodium butyrate on the viability and life span in Drosophila melanogaster]. Adv Gerontol. (2012) 25:126–31. [in Russian] 22708457

[85] Wang P Zhang Y Gong Y Yang R Chen Z Hu W. Sodium butyrate triggers a functional elongation of microglial process via Akt-small RhoGTPase activation and HDACs inhibition. Neurobiol Dis. (2018) 111:12–25. 10.1016/j.nbd.2017.12.006 29248540

[86] Ho L Ono K Tsuji M Mazzola P Singh R Pasinetti GM. Protective roles of intestinal microbiota derived short chain fatty acids in Alzheimer's disease-type beta-amyloid neuropathological mechanisms. Expert Rev Neurother. (2018) 18:83–90. 10.1080/14737175.2018.1400909 29095058 PMC5958896

[87] Hoffman JD Yanckello LM Chlipala G Hammond TC McCulloch SD Parikh I. Dietary inulin alters the gut microbiome, enhances systemic metabolism and reduces neuroinflammation in an APOE4 mouse model. PLoS ONE. (2019) 14:e0221828. 10.1371/journal.pone.0221828 31461505 PMC6713395

[88] Colombo AV Sadler RK Llovera G Singh V Roth S Heindl S. Microbiota-derived short chain fatty acids modulate microglia and promote Aβ plaque deposition. Elife. (2021) 10:e59826. 10.7554/eLife.59826 33845942 PMC8043748

[89] Marizzoni M Cattaneo A Mirabelli P Festari C Lopizzo N Nicolosi V. Short-chain fatty acids and lipopolysaccharide as mediators between gut dysbiosis and amyloid pathology in Alzheimer's disease. J Alzheimers Dis. (2020) 78:683–97. 10.3233/JAD-200306 33074224

[90] Pacheco-Ordaz R Wall-Medrano A Goñi MG Ramos-Clamont-Montfort G Ayala-Zavala JF González-Aguilar GA. Effect of phenolic compounds on the growth of selected probiotic and pathogenic bacteria. Lett Appl Microbiol. (2018) 66:25–31. 10.1111/lam.12814 29063625

[91] Moreno-Indias I Sánchez-Alcoholado L Pérez-Martínez P Andrés-Lacueva C Cardona F Tinahones F. Red wine polyphenols modulate fecal microbiota and reduce markers of the metabolic syndrome in obese patients. Food Funct. (2016) 7:1775–87. 10.1039/C5FO00886G 26599039

[92] Braune A Blaut M. Bacterial species involved in the conversion of dietary flavonoids in the human gut. Gut Microbes. (2016) 7:216–34. 10.1080/19490976.2016.1158395 26963713 PMC4939924

[93] Marín L Miguélez EM Villar CJ Lombó F. Bioavailability of dietary polyphenols and gut microbiota metabolism: antimicrobial properties. Biomed Res Int. (2015) 2015:905215. 10.1155/2015/905215 25802870 PMC4352739

[94] Shishtar E Rogers GT Blumberg JB Au R Jacques PF. Long-term dietary flavonoid intake and risk of Alzheimer disease and related dementias in the Framingham Offspring Cohort. Am J Clin Nutr. (2020) 112:343–53. 10.1093/ajcn/nqaa079 32320019 PMC7398772

[95] Cho I Song HO Cho JH. Flavonoids mitigate neurodegeneration in aged Caenorhabditis elegans by mitochondrial uncoupling. Food Sci Nutr. (2020) 8:6633–42. 10.1002/fsn3.1956 33312547 PMC7723185

[96] Khan H Ullah H Aschner M Cheang WS Akkol EK. Neuroprotective effects of quercetin in Alzheimer's disease. Biomolecules. (2019) 10:59. 10.3390/biom10010059 31905923 PMC7023116

[97] Gomes BAQ Silva JPB Romeiro CFR Dos Santos SM Rodrigues CA Gonçalves PR. Neuroprotective mechanisms of resveratrol in Alzheimer's disease: role of SIRT1. Oxid Med Cell Longev. (2018) 2018:8152373. 10.1155/2018/8152373 30510627 PMC6232815

[98] Mori T Koyama N Yokoo T Segawa T Maeda M Sawmiller D. Gallic acid is a dual α/β-secretase modulator that reverses cognitive impairment and remediates pathology in Alzheimer mice. J Biol Chem. (2020) 295:16251–66. 10.1074/jbc.RA119.012330 32913125 PMC7705308

[99] Bernardi S Del Bo' C Marino M Gargari G Cherubini A Andrés-Lacueva C. Polyphenols and intestinal permeability: rationale and future perspectives. J Agric Food Chem. (2020) 68:1816–29. 10.1021/acs.jafc.9b02283 31265272

[100] Del Bo' C Bernardi S Cherubini A Porrini M Gargari G Hidalgo-Liberona N. A polyphenol-rich dietary pattern improves intestinal permeability, evaluated as serum zonulin levels, in older subjects: the MaPLE randomised controlled trial. Clin Nutr. (2021) 40:3006–18. 10.1016/j.clnu.2020.12.014 33388204

[101] Koudoufio M Desjardins Y Feldman F Spahis S Delvin E Levy E. Insight into polyphenol and gut microbiota crosstalk: are their metabolites the key to understand protective effects against metabolic disorders? Antioxidants (Basel). (2020) 9:982. 10.3390/antiox9100982 33066106 PMC7601951

[102] Gutiérrez-Díaz I Fernández-Navarro T Sánchez B Margolles A González S. Mediterranean diet and faecal microbiota: a transversal study. Food Funct. (2016) 7:2347–56. 10.1039/C6FO00105J 27137178

[103] Papandreou C Moré M Bellamine A. Trimethylamine N-oxide in relation to cardiometabolic health-cause or effect? Nutrients. (2020) 12:1330. 10.3390/nu12051330 32392758 PMC7284902

[104] Nowiński A Ufnal M. Trimethylamine N-oxide: a harmful, protective or diagnostic marker in lifestyle diseases? Nutrition. (2018) 46:7–12. 10.1016/j.nut.2017.08.001 29290360

[105] Montgomery SA Thal LJ Amrein R. Meta-analysis of double blind randomized controlled clinical trials of acetyl-L-carnitine versus placebo in the treatment of mild cognitive impairment and mild Alzheimer's disease. Int Clin Psychopharmacol. (2003) 18:61–71. 10.1097/00004850-200303000-00001 12598816

[106] Eussen SJ Ueland PM Clarke R Blom HJ Hoefnagels WH van Staveren WA. The association of betaine, homocysteine and related metabolites with cognitive function in Dutch elderly people. Br J Nutr. (2007) 98:960–8. 10.1017/S0007114507750912 17537289

[107] Blusztajn JK Slack BE Mellott TJ. Neuroprotective actions of dietary choline. Nutrients. (2017) 9:815. 10.3390/nu9080815 PMC5579609 28788094

[108] Velazquez R Ferreira E Knowles S Fux C Rodin A Winslow W. Lifelong choline supplementation ameliorates Alzheimer's disease pathology and associated cognitive deficits by attenuating microglia activation. Aging Cell. (2019) 18:e13037. 10.1111/acel.13037 31560162 PMC6826123

[109] Kepka A Ochocinska A Borzym-Kluczyk M Skorupa E Stasiewicz-Jarocka B Chojnowska S. Preventive role of l-carnitine and balanced diet in Alzheimer's disease. Nutrients. (2020) 12:1987. 10.3390/nu12071987 32635400 PMC7400709

[110] Ibi D Hirashima K Kojima Y Sumiya K Kondo S Yamamoto M. Preventive effects of continuous betaine intake on cognitive impairment and aberrant gene expression in hippocampus of 3xTg mouse model of Alzheimer's disease. J Alzheimers Dis. (2021) 79:639–52. 10.3233/JAD-200972 33337369

[111] Simó C García-Cañas V. Dietary bioactive ingredients to modulate the gut microbiota-derived metabolite TMAO. New opportunities for functional food development. Food Funct. (2020) 11:6745–76. 10.1039/D0FO01237H 32686802

[112] Berge RK Ramsvik MS Bohov P Svardal A Nordrehaug JE Rostrup E. Krill oil reduces plasma triacylglycerol level and improves related lipoprotein particle concentration, fatty acid composition and redox status in healthy young adults - a pilot study. Lipids Health Dis. (2015) 14:163. 10.1186/s12944-015-0162-7 26666303 PMC4678523

[113] Lemos BS Medina-Vera I Malysheva OV Caudill MA Fernandez ML. Effects of egg consumption and choline supplementation on plasma choline and trimethylamine-N-oxide in a young population. J Am Coll Nutr. (2018) 37:716–23. 10.1080/07315724.2018.1466213 29764315

[114] Griffin LE Djuric Z Angiletta CJ Mitchell CM Baugh ME Davy KP. A Mediterranean diet does not alter plasma trimethylamine N-oxide concentrations in healthy adults at risk for colon cancer. Food Funct. (2019) 10:2138–47. 10.1039/C9FO00333A 30938383 PMC6552673

[115] Wang Z Bergeron N Levison BS Li XS Chiu S Jia X. Impact of chronic dietary red meat, white meat, or non-meat protein on trimethylamine N-oxide metabolism and renal excretion in healthy men and women. Eur Heart J. (2019) 40:583–94. 10.1093/eurheartj/ehy799 30535398 PMC6374688

[116] Romano KA Vivas EI Amador-Noguez D Rey FE. Intestinal microbiota composition modulates choline bioavailability from diet and accumulation of the proatherogenic metabolite trimethylamine-N-oxide. mBio. (2015) 6:e02481. 10.1128/mBio.02481-14 25784704 PMC4453578

[117] Cattaneo A Cattane N Galluzzi S Provasi S Lopizzo N Festari C. Association of brain amyloidosis with pro-inflammatory gut bacterial taxa and peripheral inflammation markers in cognitively impaired elderly. Neurobiol Aging. (2017) 49:60–8. 10.1016/j.neurobiolaging.2016.08.019 27776263

[118] Cho CE Taesuwan S Malysheva OV Bender E Tulchinsky NF Yan J. Trimethylamine-N-oxide (TMAO) response to animal source foods varies among healthy young men and is influenced by their gut microbiota composition: a randomized controlled trial. Mol Nutr Food Res. (2017) 61:1600324. 10.1002/mnfr.201770016 27377678

[119] Wu WK Chen CC Liu PY Panyod S Liao BY Chen PC. Identification of TMAO-producer phenotype and host-diet-gut dysbiosis by carnitine challenge test in human and germ-free mice. Gut. (2019) 68:1439–49. 10.1136/gutjnl-2018-317155 30377191 PMC6691853

[120] Chung SJ Rim JH Ji D Lee S Yoo HS Jung JH. Gut microbiota-derived metabolite trimethylamine N-oxide as a biomarker in early Parkinson's disease. Nutrition. (2021) 83:111090. 10.1016/j.nut.2020.111090 33418492

[121] Chen L Chen Y Zhao M Zheng L Fan D. Changes in the concentrations of trimethylamine N-oxide (TMAO) and its precursors in patients with amyotrophic lateral sclerosis. Sci Rep. (2020) 10:15198. 10.1038/s41598-020-72184-3 32938991 PMC7495434

[122] Pluta R Ułamek-Kozioł M Januszewski S Czuczwar SJ. Gut microbiota and pro/prebiotics in Alzheimer's disease. Aging (Albany NY). (2020) 12:5539–50. 10.18632/aging.102930 32191919 PMC7138569

[123] Arora K Green M Prakash S. The microbiome and Alzheimer's disease: potential and limitations of prebiotic, synbiotic, and probiotic formulations. Front Bioeng Biotechnol. (2020) 8:537847. 10.3389/fbioe.2020.537847 33384986 PMC7771210

[124] Liu Q Xi Y Wang Q Liu J Li P Meng X. Mannan oligosaccharide attenuates cognitive and behavioral disorders in the 5xFAD Alzheimer's disease mouse model via regulating the gut microbiota-brain axis. Brain Behav Immun. (2021) 95:330–43. 10.1016/j.bbi.2021.04.005 33839232

[125] Xin Y Diling C Jian Y Ting L Guoyan H Hualun L. Effects of oligosaccharides from Morinda officinalis on gut microbiota and metabolome of APP/PS1 transgenic mice. Front Neurol. (2018) 9:412. 10.3389/fneur.2018.00412 29962999 PMC6013575

[126] Han D Li Z Liu T Yang N Li Y He J. Prebiotics regulation of intestinal microbiota attenuates cognitive dysfunction induced by surgery stimulation in APP/PS1 mice. Aging Dis. (2020) 11:1029–45. 10.14336/AD.2020.0106 33014520 PMC7505279

[127] Xu M Mo X Huang H Chen X Liu H Peng Z. Yeast β-glucan alleviates cognitive deficit by regulating gut microbiota and metabolites in Aβ1-42-induced AD-like mice. Int J Biol Macromol. (2020) 161:258–70. 10.1016/j.ijbiomac.2020.05.180 32522544

[128] Lee YS Lai DM Huang HJ Lee-Chen GJ Chang CH Hsieh-Li HM. Prebiotic lactulose ameliorates the cognitive deficit in Alzheimer's disease mouse model through macroautophagy and chaperone-mediated autophagy pathways. J Agric Food Chem. (2021) 69:2422–37. 10.1021/acs.jafc.0c07327 33617267

[129] Kobayashi Y Sugahara H Shimada K Mitsuyama E Kuhara T Yasuoka A. Therapeutic potential of Bifidobacterium breve strain A1 for preventing cognitive impairment in Alzheimer's disease. Sci Rep. (2017) 7:13510. 10.1038/s41598-017-13368-2 29044140 PMC5647431

[130] KobayashiYKinoshitaTMatsumotoAYoshinoKSaitoIXiaoJZ. Bifidobacterium breve A1 supplementation improved cognitive decline in older adults with mild cognitive impairment: an open-label, single-arm study. J Prev Alzheimers Dis. (2019) 6:70–5. 10.14283/jpad.2018.3230569089

[131] Xiao J Katsumata N Bernier F Ohno K Yamauchi Y Odamaki T. Probiotic Bifidobacterium breve in improving cognitive functions of older adults with suspected mild cognitive impairment: a randomized, double-blind, placebo-controlled trial. J Alzheimers Dis. (2020) 77:139–47. 10.3233/JAD-200488 32623402 PMC7592675

[132] Leblhuber F Steiner K Schuetz B Fuchs D Gostner JM. Probiotic supplementation in patients with Alzheimer's dementia - an explorative intervention study. Curr Alzheimer Res. (2018) 15:1106–13. 10.2174/1389200219666180813144834 30101706 PMC6340155

[133] Ton AMM Campagnaro BP Alves GA Aires R Côco LZ Arpini CM. Oxidative stress and dementia in Alzheimer's patients: effects of synbiotic supplementation. Oxid Med Cell Longev. (2020) 2020:2638703. 10.1155/2020/2638703 32411323 PMC7201593

[134] Tamtaji OR Heidari-Soureshjani R Mirhosseini N Kouchaki E Bahmani F Aghadavod E. Probiotic and selenium co-supplementation, and the effects on clinical, metabolic and genetic status in Alzheimer's disease: a randomized, double-blind, controlled trial. Clin Nutr. (2019) 38:2569–75. 10.1016/j.clnu.2018.11.034 30642737

[135] Vendrik KEW Ooijevaar RE de Jong PRC Laman JD van Oosten BW van Hilten JJ. Fecal microbiota transplantation in neurological disorders. Front Cell Infect Microbiol. (2020) 10:98. 10.3389/fcimb.2020.00098 32266160 PMC7105733

[136] Kim MS Kim Y Choi H Kim W Park S Lee D. Transfer of a healthy microbiota reduces amyloid and tau pathology in an Alzheimer's disease animal model. Gut. (2020) 69:283–94. 10.1136/gutjnl-2018-317431 31471351

[137] Hazan S. Rapid improvement in Alzheimer's disease symptoms following fecal microbiota transplantation: a case report. J Int Med Res. (2020) 48:300060520925930. 10.1177/0300060520925930 32600151 PMC7328362

